# Recombination Variation Shapes Phylogeny and Introgression in Wild Diploid Strawberries

**DOI:** 10.1093/molbev/msad049

**Published:** 2023-03-02

**Authors:** Chao Feng, Jing Wang, Aaron Liston, Ming Kang

**Affiliations:** Key Laboratory of Plant Resources Conservation and Sustainable Utilization, South China Botanical Garden, Chinese Academy of Sciences, Guangzhou, China; Guangdong Provincial Key Laboratory of Applied Botany, South China Botanical Garden, Chinese Academy of Sciences, Guangzhou, China; Institute of Pomology, Jiangsu Academy of Agricultural Sciences/Jiangsu Key Laboratory for Horticultural Crop Genetic Improvement, Nanjing, China; Department of Botany and Plant Pathology, Oregon State University, Corvallis, OR; Key Laboratory of Plant Resources Conservation and Sustainable Utilization, South China Botanical Garden, Chinese Academy of Sciences, Guangzhou, China; Guangdong Provincial Key Laboratory of Applied Botany, South China Botanical Garden, Chinese Academy of Sciences, Guangzhou, China

**Keywords:** *Fragaria*, introgression, mating system, phylogenomics, recombination rate, wild diploid strawberry

## Abstract

Introgressive hybridization is widespread in wild plants and has important consequences. However, frequent hybridization between species makes the estimation of the species’ phylogeny challenging, and little is known about the genomic landscape of introgression as it results from complex interactions of multiple evolutionary processes. Here, we reconstructed the phylogeny of ten wild diploid strawberries with whole genome resequencing data and then investigated the influence of recombination rate variation on phylogeny and introgression. We found that genomic regions with low recombination showed reduced levels of incomplete lineage sorting and introgression, and concentrated phylogenetic signals, thus contributing to the most likely species tree of wild diploid strawberries. We revealed complex and widespread introgression across the genus *Fragaria*, with an average proportion of approximately 4.1% of the extant genome. Introgression tends to be retained in the regions with high recombination rates and low gene density. Furthermore, we identified four *SLF* genes under selective sweeps that may play potential roles in the possible regain of self-incompatibility by ancient introgression. Altogether, our study yielded novel insights into the evolutionary history and genomic characteristics of introgression in wild diploid strawberries and provides evidence for the role of introgression in plant mating system transitions.

## Introduction

Hybridization is quite common and an important force in plant evolution, with many plant species showing hybrid origins (Mallet 2005; Soltis and Soltis 2009). One of its outcomes is introgression—the transfer of genetic material between or within species by hybridization and repeated backcrossing. This process is believed to be widespread in nature, as revealed by recent genome-scale sequence data (Mallet et al. 2016; Edelman and Mallet 2021; Moran et al. 2021). As such, introgressive hybridization has been detected in many groups of species, including Arabidopsis (Arnold et al. 2016), monkeyflowers (Stankowski and Streisfeld 2015), tomatoes (Pease et al. 2016), *Drosophila* (Suvorov et al. 2022), butterflies (Martin et al. 2013; Edelman et al. 2019; Kozak et al. 2021), lizards (Finger et al. 2022), birds (Ellegren et al. 2012; Singhal et al. 2021), mammals (Jones et al. 2018; Shi and Yang 2018; Ferreira et al. 2021), and hominins (Nielsen et al. 2017). These studies have considerably improved our understanding of the role of hybridization and introgression in nature (Payseur and Rieseberg 2016; Martin and Jiggins 2017; Edelman and Mallet 2021). However, it remains a challenge to characterize the genomic landscape of introgression as it results from complex interactions of multiple evolutionary processes (e.g., selection, demography, and recombination) (Martin and Jiggins 2017).

Following hybridization, recombination can break up the linkage between alleles at different loci, thereby generating novel combinations across loci that can be exposed to selection (Schumer et al. 2018). As a result, regions of the genome with high recombination can more rapidly decouple neutral and adaptive introgressed alleles from deleterious alleles and, therefore, tend to harbor a higher proportion of introgression, whereas a localized reduction in introgression is expected within low recombination regions due to the increased linkage between introgressed loci and neighboring selected variants (Nachman and Payseur 2012; Schumer et al. 2018; Martin et al. 2019). Therefore, recombination rate variation is expected to play an important role in mediating the efficacy of selection and shaping patterns of introgression across the genome. However, how recombination rate variation impacts the genomic landscape of introgression remains an open question despite the increasing attention it has received in the last few years (Martin and Jiggins 2017; Kim et al. 2018; Schumer et al. 2018; Leitwein et al. 2019; Martin et al. 2019; Owens et al. 2022).

Reconstructing the history of hybridization and introgression in any evolutionary radiation requires a robust backbone of phylogenetic relationships among species. The recent emergence of phylogenomic data sets with hundreds or thousands of loci provides unprecedented opportunities to resolve the evolutionary history of species. However, phylogenetic trees based on genome-wide sequence data may not always represent the true, species-level relationships. Gene tree heterogeneity is widespread across the genome and often poses significant challenges for phylogenetic inference as a result of two primary processes, incomplete lineage sorting (ILS) and gene flow (Degnan and Rosenberg 2009). Although several methods have been developed and can explicitly accommodate ILS as the source of discordance (Edwards et al. 2016; Mirarab et al. 2016; Xu and Yang 2016), identifying and accounting for other processes such as gene flow in empirical datasets remain challenging (Li et al. 2019), particularly for lineages with an extensive history of hybridization and introgression, which in turn makes it difficult to infer the history of hybridization and introgression. As a key genetic parameter that influences assessments of gene flow, ILS, and genetic diversity, recombination rate variation plays a critical role in shaping the distribution of phylogenetic signals. The recombination rate interacts with the effects of natural selection to influence how genealogical histories are distributed across the genome. As mentioned above, low recombination regions of the genome are generally depleted in signatures of hybridization and are typically enriched for the most likely species tree (Pease and Hahn 2013). However, recombination rate variation among markers is not considered in most phylogenetic studies, and only a few empirical studies have attempted to directly quantify the impact of recombination rate variation on phylogenetic inferences (Pease and Hahn 2013; Edelman et al. 2019; Li et al. 2019; Martin et al. 2019; Hennelly et al. 2021; Owens et al. 2022). Therefore, we still have a poor understanding of the impact of recombination on species tree reconstructions.

The genus *Fragaria* contains approximately 25 species in five even-ploidy levels, ranging from diploid to decaploid with a basic chromosome number of seven (Folta and Davis 2006; Hummer and Hancock 2009; Liston et al. 2014). Wild *Fragaria* have a distribution spanning the Northern Hemisphere with the center of diversity being within China, where the majority of diploid (9 out of 12) and all five tetraploid species of the genus occur (Liston et al. 2014; Lei et al. 2017). The mating system varies substantially across species in *Fragaria*, from self-compatibility (SC), through self-incompatibility (SI), to dioecy (Liston et al. 2014). Moreover, *Fragaria* species are known to have small genomes (200–300 Mb for diploid species) and are amenable to propagation in tissue culture and genetic transformation. These characteristics render *Fragaria* an emerging model system for studies of sexual system evolution, polyploidization, and evolutionary genomics (Liston et al. 2014).

The phylogenetic relationships among species of *Fragaria* remain unresolved despite numerous efforts using chloroplast genomes (Njuguna et al. 2013), mitochondrial genomes (Fan et al. 2022), target capture sequencing (Kamneva et al. 2017), RNASeq (Qiao et al. 2016), multi-locus genes (Yang and Davis 2017), and the sequencing of whole genomes (Feng et al. 2021; Qiao et al. 2021). A major challenge in reconstructing the *Fragaria* phylogeny is the rapid radiation process, which is accompanied by both extensive ILS and hybridization between nascent lineages, leading to extensive gene-tree heterogeneity across the genome. For example, Feng et al. (2021) reconstructed a genome-wide phylogeny of five diploid species with 8,663 single-copy genes and revealed extensive gene–tree discordance, due to both extensive ILS and interspecific hybridization. More recently, Qiao et al. (2021) conducted the phylogenetic analysis of ten diploid species based on concatenation analysis of 1,007 single-copy genes with a maximum likelihood (ML) method, but the effect of neither ILS nor gene flow on phylogenetic reconstruction was accounted for in the analysis. As a result, our understanding of the full speciation history of *Fragaria* remains limited. Furthermore, natural hybridization and introgression between species are well-documented within *Fragaria* (Bringhurst and Khan 1963; Bringhurst and Senanayake 1966; Staudt 1999; Lei et al. 2005). However, the spatial landscape of introgression across the genome and its association with recombination and gene density are poorly understood.

The aim of the present study is to reconstruct phylogenetic relationships of wild diploid strawberries and characterize the genomic landscape of introgression among species. To this end, we generated whole-genome resequencing data of 68 wild diploid strawberries representing ten *Fragaria* species and reconstructed phylogenetic trees with partitioned recombination rates to investigate the relationships between recombination rates and frequencies of particular topologies. We then integrated a series of population genetic and phylogenetic approaches to assess hybridization patterns and the rate of gene flow between taxa. In particular, we performed a detailed characterization of genomic introgression in terms of recombination rate and gene density. Finally, we carried out a selective sweep analysis to identify regions and genes under selection in wild diploid strawberries and discuss their potential role in mating system transitions.

## Results

### Phylogeny and Population Structure

We sequenced and analyzed the genomes of 68 wild diploid strawberries representing ten *Fragaria* species, *F. chinensis*, *F. nipponica*, *F. nubicola*, *F. pentaphylla*, *F. nilgerrensis*, *F. daltoniana*, *F. iinumae*, *F. viridis*, *F. vesca*, and *F. mandshurica*, with a mean depth of approximately 54× per individuals covering an average of approximately 86% of the reference genome (supplementary table S1, Supplementary Material online). Along with one sample from the outgroup *Potentilla microphylla* (Buti et al. 2018), we identified approximately 14 million high-quality single-nucleotide polymorphisms (SNPs) within and across species.

**Fig. 1.**
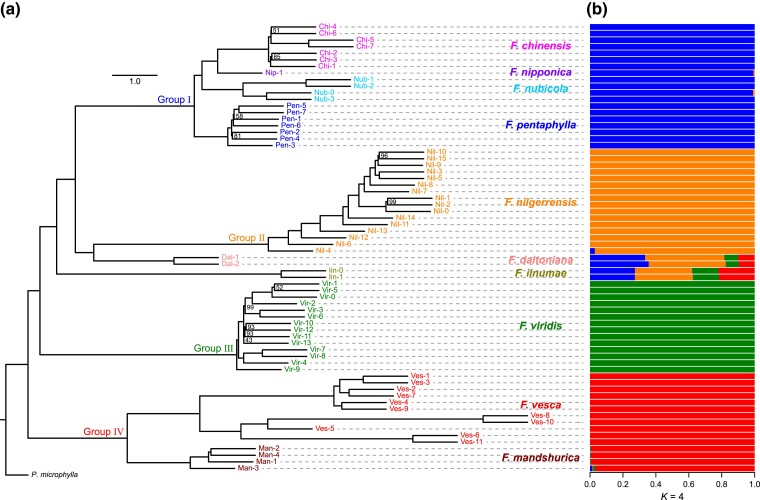
The phylogenetic tree and genetic structure of the ten wild diploid strawberries. (*a*) The ASTRAL tree for the 68 individuals representing the ten wild diploid strawberry species from low recombination regions, setting *P. microphylla* as the outgroup. The tree is constructed using 3,859 ML trees from SNPs of 10 kb nonoverlapping sliding windows with the 20% lowest recombination rate across the genome. The bootstrap support values less than 100% are shown along the nodes. (*b*) The optimal genetic structure of the 68 wild diploid strawberries detected by the Structure analysis (*K* = 4) based on genome-wide unlinked SNPs.

We created a concatenation ML tree and a coalescent-based summary ASTRAL tree at the whole genome level, chromosome scales, and genomic windows with partitioned recombination rate, respectively (fig. 1*a*, supplementary fig. S1, Supplementary Material online and supplementary table S2, Supplementary Material online). In addition, we estimated a site-based coalescent SVDQuartets tree with reduced SNPs across the genome and built the whole chloroplast genome tree (supplementary fig. S1, Supplementary Material online). Although ML, ASTRAL, and SVDQuartets trees had strong support (100%) for nodes that separated species groups, these phylogenetic reconstructions were inconsistent with each other (supplementary fig. S1, Supplementary Material online) and showed strong discordance with the phylogenetic relationship of species trees from previous studies (supplementary fig. S2, Supplementary Material online; Njuguna et al. 2013; Kamneva et al. 2017; Qiao et al. 2016, 2021; Edger et al. 2019; Liston et al. 2020; Fan et al. 2022). All these phylogenetic trees support that *F. vesca* and *F. mandshurica* comprise one clade, while in most studies, several species native to Southwest China, *F. chinensis*, *F. nubicola*, *F. pentaphylla*, *F. nilgerrensis*, and *F. daltoniana*, are grouped into another clade (designated as Clade Southwest China). The main discordance of these phylogenies involves the phylogenetic relationships of *F*. *iinumae* and *F. viridis.* The phylogenetic trees inferred by Kamneva et al. (2017), Edger et al. (2019), and Liston et al. (2020), and this study clearly indicates that *F*. *iinumae* is sister to the Clade Southwest China (supplementary fig. S2, Supplementary Material online), while the species tree inferred from genome-wide orthologous single-copy genes (Qiao et al. 2021) and mitochondrial genome (Fan et al. 2022) support *F*. *iinumae* as the first-diverging lineage among diploid strawberry (supplementary fig. S2, Supplementary Material online). Additionally, two coalescent trees (ASTRAL and SVDQuartets tree) inferred from the whole genome and ASTRAL/ML tree inferred from low recombination regions in this study illustrate that *F. viridis* is a sister to the Clade Southwest China and *F*. *iinumae* (supplementary figs. S1 and S2, Supplementary Material online), while the ML tree constructed by concatenation sites across the whole genome, plastome trees (Njuguna et al. 2013), mitochondrial genome tree (Fan et al. 2022), and several other studies (Kamneva et al. 2017; Edger et al. 2019; Liston et al. 2020; Qiao et al. 2021) show that *F. viridis* is sister to the Clade of *F. vesca* and *F. mandshurica* (supplementary figs. S1 and S2, Supplementary Material online). Furthermore, the relative phylogenetic relationships among *F. chinensis*, *F. nipponica*, *F. pentaphylla*, and *F. nubicola* are heterogeneous (supplementary figs. S1 and S2, Supplementary Material online).

We investigated population structure in *Fragaria* using STRUCTURE and principal component analysis (PCA) analyses (fig. 1*b* and supplementary fig. S3, Supplementary Material online). The Δ*K* analysis indicates that the best *K* value was *K* = 4 (supplementary fig. S3, Supplementary Material online). For *K* = 4, the individuals were generally separated into four groups corresponding to the four phylogenetic lineages, while two species, *F. daltoniana* and *F. iinumae*, showed a high degree of admixture (fig. 1*b*), indicating potential pervasive hybridization or introgression. The population genetic structure with *K* ranging from 2 to 5 is also shown in supplementary figure S3, Supplementary Material online to fully explore population subdivision. The individual ancestry assignment estimated by STRUCTURE (*K* = 4) is highly consistent with the PCA results (supplementary fig. S3, Supplementary Material online), where the first three principal components contributed nearly half (46.9%) of total genetic variance and clearly distinguished these four groups and two admixed species (*F. daltoniana* and *F. iinumae*). For example, group III (*F. viridis*) and group IV (*F. mandshurica* and *F. vesca*) were independent of each other along the PC2, while group II (*F. nilgerrensis*) separated from other groups according to PC3 (supplementary fig. S3, Supplementary Material online).

### Topology Weighting Reveals Phylogenetic Discordance

**Fig. 2.**
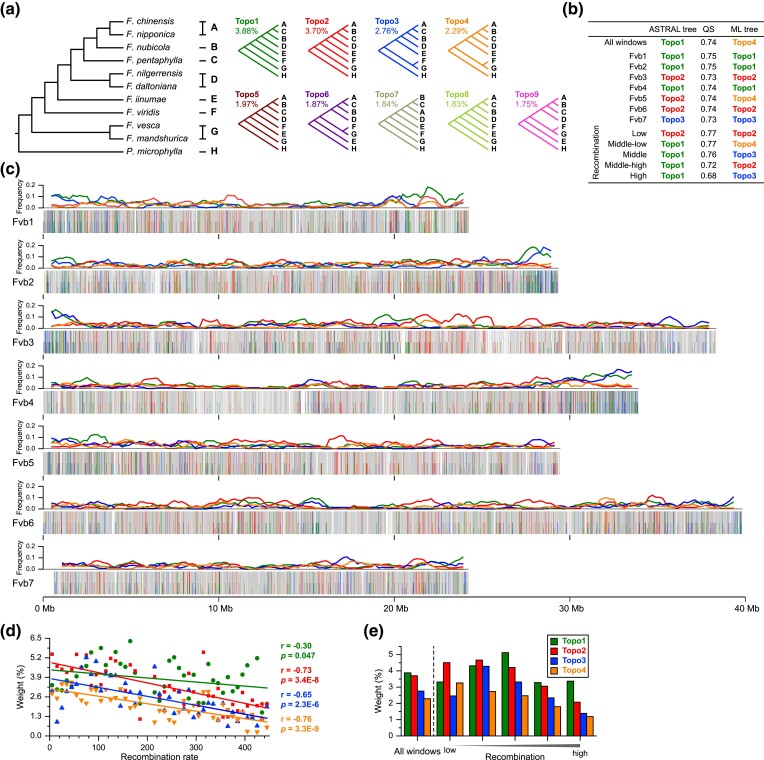
Variation of topology weighting within and among chromosomes reveals widespread phylogenetic discordance and is correlated with recombination rate. (*a*) The main possible topologies, with occurrence frequency over 1.5%, for the eight taxa groups. (*b*) Topology for ASTRAL trees and ML tree at the whole genome level, chromosome scales, and groups with partitioned recombination rate. The normalized quartet score (QS) values reflect the level of phylogenetic discordance among gene trees inferred from corresponding windows. (*c*) Frequency distribution of four main topologies (colors as in panel a) for 500 kb sliding windows with the step size of 100 kb (Upper line chart), and weighting for the four main possible topologies (colors as in panel *a*) plotted across 10 kb nonoverlopping sliding windows (Lower bar chart) along the chromosomes, where grey and white columns indicate the windows belonging to other topologies, and the windows with unknown topology, respectively. (*d*) Average weightings for the top four possible topologies (colors as in panel *a*) binned according to their recombination rate and the linear regression curve. The *r* (Pearson Correlation Coefficient) and *P* values are shown as the colors of the corresponding topology in panel (*a*). (*e*) Frequency of the top four possible topologies at the whole genome level and five groups with partitioned recombination rate.

To explore phylogenetic conflicts along the chromosomes, we calculated the frequency of topology under eight taxa combinations (see Materials and Methods) for each of the 10 kb nonoverlapping sliding windows (supplementary table S3, Supplementary Material online). There are nine main topologies with occurrence frequency over 1.5% at the whole genome level, the top four of which have topology weighting ranging from 2.2% to 3.9% (fig. 2*a* and supplementary table S4, Supplementary Material online). Topo1 and Topo2 are the phylogenetic hypotheses inferred from ASTRAL with the whole genome and low recombination windows, respectively (fig. 2*a* and *b*, supplementary fig. S1, Supplementary Material online and supplementary table S4, Supplementary Material online). Topo3 and Topo4 correspond to Topo1 and Topo2, respectively, with the sole change in the phylogenetic position of *F. viridis* as the sister group of *F. vesca* and *F. mandshurica*, instead of the sister group of Clade Southwest China and *F. iinumae* (fig. 2*a*). The main topologies do not appear to be randomly distributed across the genome. Topo1 and Topo3 tend to be located at the end of chromosomes, while Topo2 is enriched in the center of chromosomes (fig. 2*c*). It is worth noting that Topo2 has the highest topology weighting and is consistent with both the ASTRAL and ML trees inferred with low recombination windows and the two longest chromosomes (Fvb3 and Fvb6; fig. 2*b* and supplementary table S4, Supplementary Material online). We further investigated the genomic characteristics of the main topologies and found that the weightings of the top four abundant topologies were significantly reduced with the increase of recombination rate (fig. 2*d*). Although Topo1 has a slightly higher average frequency compared to Topo2 at the whole genome level, Topo 2 occurs more frequently in low recombination regions (fig. 2*e* and supplementary table S4, Supplementary Material online).

The normalized quartet score is relatively high (0.77) for the species tree inferred from low/medium-low recombination windows compared to that for high recombination regions (0.68), indicating a lower level of gene tree discordance in regions of low recombination. Additionally, the MSCquartets analyses show less blue circles plotted close to centroids of the simplexes in the trees inferred from low recombination windows, compared to the trees from high recombination regions (supplementary fig. S4, Supplementary Material online), which indicates that low recombination regions tend to contain less ILS. As a result, we expect that the topology resulting from low recombination regions should be more likely to reflect the true phylogeny of the wild diploid strawberries.

### Widespread Introgression Among Species

**Fig. 3.**
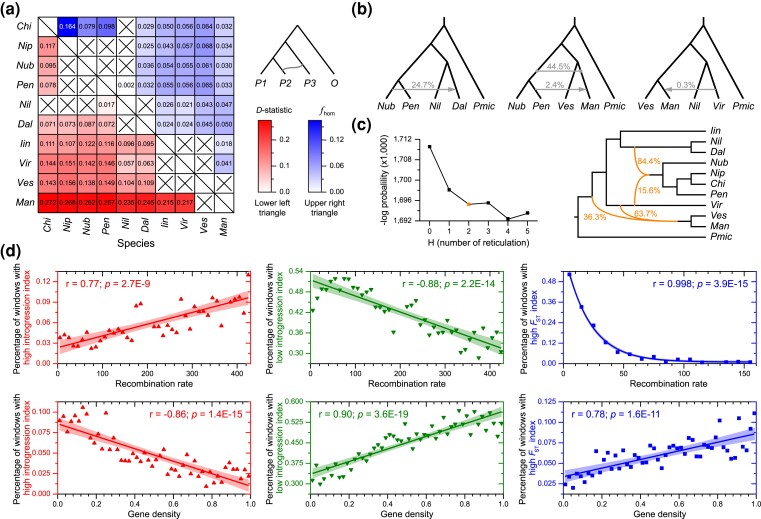
The widespread introgression across wild diploid strawberry genomes and its association with recombination rate and gene density. (*a*) Heatmap of significantly elevated *D*_min_ score (Lower left triangular matrix) and genomic properties of introgression (*f*_hom_ value, Upper right triangular matrix) between *P2* and *P3*. The color of the corresponding heatmap cell represents the most significant *D*_min_ score with adjusted *P* value <0.01 and the maximum *f*_hom_ value across all possible species in *P1*. (*b*) Signals of introgression in *Fragaria* inferred by *D*_FOIL_ analyses based on five-taxon phylogeny (((*P1*, *P2*), (*P3*, *P4*)), *O*), where the divergence time between *P3* and *P4* was earlier than that between *P1* and *P2*. The horizontal line with double-ended dots and lines with single-ended arrows indicate ancient introgression and post-speciation gene flow, respectively. The numbers above the lines show the proportion of individual combinations that detected significant signals of introgression for the corresponding species combinations. (*c*) Signals of gene flow in wild diploid *Fragaria* species inferred by PhyloNet. The numbers next to the lines indicate inheritance probabilities for corresponding edges. (*d*) Average percentage of windows with high ingression index, low ingression index, and high genetic differentiation (*F*_ST_) index binned according to their recombination rate and gene density. The *r* (Pearson Correlation Coefficient) and *P* values are shown along the linear regression curve. *Chi*, *F. chinensis*; *Nip*, *F. nipponica*; *Nub, F. nubicola*; *Pen*, *F. pentaphylla*; *Nil*, *F. nilgerrensis*; *Dal*, *F. daltoniana*; *Iin*, *F. iinumae*; *Vir*, *F. viridis*; *Ves*, *F. vesca*; *Man*, *F. mandshurica*; *Pmic*, *P. microphylla*.

ABBA-BABA tests revealed that about 79% (95/120) of tested four-taxon phylogenies had a significant signal of introgression (*P* < 0.01 after Benjamini–Hochberg correction) (fig. 3*a* and supplementary table S5, Supplementary Material online), indicating widespread hybridization and introgression history in wild diploid *Fragaria* species. The *D* statistic is widely used in detecting introgression but is unable to estimate the proportion of the genome with evidence of introgression (Martin et al. 2015; Malinsky et al. 2021; Morales-Cruz et al. 2021). To further determine the proportion of the genome with evidence of introgression, we calculated *f*_hom_ value of 95 trios with significant introgression signals, as an alternative estimator of genome-wide fraction of admixture (Pulido-Santacruz et al. 2020). As the estimates of *f*_hom_ value for the same *P2*-*P3* species pairs varied on different *P1* species (supplementary table S5, Supplementary Material online), we used their maximum values to reflect the genomic proportion of introgression between *P2* and *P3* species, remaining maximum *f*_hom_ values of 49 *P2*-*P3* species pairs (supplementary table S5, Supplementary Material online). Overall, the average extent of introgression in *Fragaria* is approximately 4.1% of the genome, comprising up to approximately 16.4% of the extant genome in comparisons between *F. nipponica* (*P2*) and *F. chinensis* (*P3*) (fig. 3*a* and supplementary table S5, Supplementary Material online). The extensive genomic introgression detected between *F. nipponica* and *F. chinensis* could explain the closer phylogenetic relationship of the two species.

We performed *D*_FOIL_ to evaluate 34 alternative symmetric five-taxon phylogenies and found signals for introgression at three species combinations, including ancient introgression and three instances of post-speciation gene flow (fig. 3*b*). The ancient introgression signal was detected between *F. mandshurica* and the ancestor of *F. nubicola* and *F. pentaphylla* (i.e., the ancestor of the lineage of *F. chinensis*, *F. nipponica*, *F. nubicola*, and *F. pentaphylla*) at a relatively high proportion (44.5%) of individual combinations (fig. 3*b*). Since a small number of ancient introgression events could create the impression of more widespread recent introgression, widespread introgression inferred by *D* statistic may be over-inflated by possible effects of early ancient introgression. In addition, nearly a quarter (24.7%) of individual combinations showed significant signals for post-speciation gene flow from *F. nubicola* to *F. daltoniana* (fig. 3*b*), which is consistent with the high degree of admixture of *F. daltoniana* in the STRUCTURE analysis (fig. 1*b*). We also estimated the substitution rates along the phylogeny at low recombination regions and observed relatively low variation among species, and no significant difference (*P* = 0.36) between SI and SC species (supplementary fig. S5, Supplementary Material online). Nevertheless, further research is needed to better understand the impact of substitution rate variation on the assessment of introgression in wild diploid strawberries.

We further applied two phylogeny-based approaches to assess the influence of hybridization and introgression on topological discordance. PhyloNet detected at least two ancient hybridizations/introgression in *Fragaria*, showing *F. viridis* may have contributed to hybrid origination of the lineage of *F. chinensis*, *F. nipponica*, *F. nubicola*, and *F. pentaphylla,* and/or the lineage of *F. vesca* and *F. mandshurica* (fig. 3*c*), which could explain the conflicting phylogenetic positions of *F. viridis* (fig. 2*a* and *b*, supplementary figs. S1 and S2, Supplementary Material online, and supplementary tables S2 and S3, Supplementary Material online) and mixture state of *F. viridis* when *K* = 2 and 3 in STRUCTURE result (supplementary fig. S3, Supplementary Material online). Limited by the requirement of a symmetric five-taxon phylogeny, partial ancient introgression events cannot be discovered in *D*_FOIL_ software, for example, the possibility of ancient introgression between *F. viridis* and the ancestor of the lineage of *F. chinensis*, *F. nipponica*, *F. nubicola*, and *F. pentaphylla* would be omitted, because *F. viridis* cannot be set as *P3*/*P4* in respective species combinations. However, our PhyloNet analyses hypothesize ancient introgression from *F. viridis* to the ancestor of the lineage of *F. chinensis*, *F. nipponica*, *F. nubicola*, and *F. pentaphylla* (fig. 3*c*), which provides an informative supplement to the *D*_FOIL_ analyses. The PhyloNetworks also identified complex hybridization and introgression in the lineage of *F. chinensis*, *F. nipponica*, *F. pentaphylla*, and *F. nubicola* (supplementary fig. S6, Supplementary Material online), which is consistent with phylogenetic discordance in our above analyses (supplementary figs. S1 and S2, Supplementary Material online). Together, these results support complex and widespread introgression in the *Fragaria* genus, and introgression substantially contributed to phylogenetic discordance.

### Genomic Landscape of Introgression is Shaped by Recombination Rate and Gene Density

We calculated the *f*_dM_ of each 10 kb nonoverlapping window for 95 trios with significant introgression signals and detected 15 to 2,749 putatively introgression regions (pIR) from each trio (supplementary table S6, Supplementary Material online), yielding 873 high introgression windows and 7,528 low introgression windows, respectively (supplementary table S7, Supplementary Material online), according to introgression index that was estimated by the proportion of trios with pIRs for each window (see Materials and Methods). Additionally, we identified 1,151 high genetic differentiation windows (introgression barrier regions) (supplementary table S8, Supplementary Material online), on a basis of the *F*_ST_ index of each window that was calculated as the proportion of combinations with top 5% *F*_ST_ values (see Materials and Methods). The genomic distribution of high/low introgression regions is a mosaic across the genome (supplementary fig. S7, Supplementary Material online), whereas the high genetic differentiation regions tend to be concentrated in the center of chromosomes (supplementary fig. S8, Supplementary Material online). Nevertheless, genomic regions with high differentiation (*F*_ST_) mainly overlapped with low and middle-low introgression regions (supplementary fig. S9, Supplementary Material online). The genes in high introgression regions are enriched for transcription regulation (supplementary table S9, Supplementary Material online), while genes in low introgression regions and high differentiation regions largely comprise specific enzyme activity (supplementary tables S10 and S11, Supplementary Material online). Worth noting, we observed that the introgression proportion of the *Fragaria* genome has an extremely strong positive relationship with recombination rate and a highly significant negative correlation with gene density (fig. 3*d*). Genomic windows of high differentiation exhibited similar characteristics with genomic windows of low introgression in that they showed a significantly negative and positive correlation with recombination rate and gene density, respectively (fig. 3*d*).

In addition, the main topology weighting reduced dramatically in the regions of high introgression and increased continually in the low introgression windows (supplementary table S12, Supplementary Material online), which indicates that introgression could influence topology weighing and phylogeny. More specifically, Topo2 has a much higher frequency than Topo1 in high genetic differentiation regions (introgression barriers) (supplementary table S12, Supplementary Material online), further providing evidence that Topo2 might reflect the most likely species tree of the *Fragaria* genus.

### Mating System Transitions May be Associated With Selective Sweeps and Introgressed Regions

**Fig. 4.**
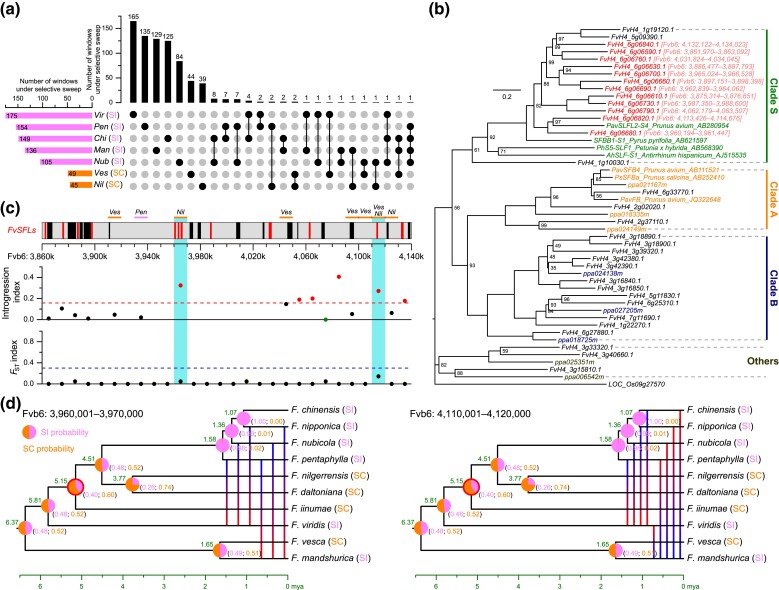
Four *S* locus-related *FvSLFs*, which are located in selective sweep and high introgression regions, may influence mating system transitions in wild diploid strawberries. (*a*) The upset plot of windows under selective sweeps of the seven *Fragaria* species. (*b*) ML tree of *SLF*/*SLF-like F-box* genes from the *F. vesca* genome and partial *F-box* genes from peach (prefix of “ppa”) and cherry. The bootstrap support values less than 100% are shown along the nodes. Clades A, B, and S are defined according to the previous phylogenetic tree of the peach genome (Akagi et al. 2016). (*c*) Gene structure, introgression, and *F*_ST_ statistics along the 280 kb putative *S* locus containing 12 *FvSLFs* in tandem duplications. The distribution of *FvSLFs* and other genes in this region is signed with red and black columns, respectively. Eight selective sweeps (10 kb window) in specific species were marked with the horizontal lines, with the species names shown above the line. (*d*) The probability of ancestral mating system state across *Fragaria* genus. The numbers in the brackets indicate the relative probabilities of SI and SC. The vertical lines indicate the introgression between *P3* and *P2* species in two windows. *Vir*, *F. viridis*; *Pen*, *F. pentaphylla*; *Chi*, *F. chinensis*; *Man*, *F. mandshurica*; *Nub*, *F. nubicola*; *Ves*, *F. vesca*; *Nil*, *F. nilgerrensis*.

Positive selection enhances adaptive evolution and leaves distinct signatures across the genome. We identified genomic regions under selective sweeps by using selscan (Szpiech and Hernandez 2014) and RAiSD (Alachiotis and Pavlidis 2018) approaches for each *Fragaria* species separately, leaving the overlapped regions regarded as the selective sweep regions for the corresponding strawberry. We screened a total of 765 windows under selective sweep for the seven wild diploid strawberries (supplementary table S13, Supplementary Material online). Two SC species, *F. nilgerrensis* and *F. vesca*, have 45 and 49 selective sweeps, respectively, which was much smaller than the number of the selective sweeps in SI species, ranging from 105 to 175 (fig. 4*a* and supplementary table S13, Supplementary Material online). Of the 765 windows, approximately 94.2% were identified under selective sweeps in a single species, while approximately 5.8% were shared by at least two species (fig. 4*a* and supplementary table S13, Supplementary Material online), indicating relatively unique adaptive patterns for each species. This conclusion is also supported by differentiated GO enrichment (specific metabolic process and enzyme activity) for genes located in selective sweeps of different diploid strawberries (supplementary table S14, Supplementary Material online).

Wild diploid strawberries have served as an important model for studying mating system transitions, due to their relatively recent origin and variation in SI and SC (Njuguna et al. 2013; Liston et al. 2014). Model comparison (symmetric vs. asymmetric) of mating system transition rates reveals that bidirectional evolution may give more reliable results (supplementary table S15, Supplementary Material online). To evaluate this, we reconstructed the probability of the ancestral type of mating system across the *Fragaria* genus by BayesTraits in RASP 4 (Yu et al. 2020). The common ancestral state of the lineages of *F. chinensis*, *F. nipponica*, *F. nubicola*, *F. pentaphylla*, *F. nilgerrensis*, *F. daltoniana*, and *F. iinuma* was SC with a probability of 60% (fig. 4*d*). Although this probability is not very high due to the small number of species involved in this study, it still indicates the possible regain of SI along the ancestor of the lineage of *F. chinensis*, *F. nipponica*, *F. nubicola*, and *F. pentaphylla*. To reveal the genetic mechanism of mating system transition in wild diploid strawberries, we focused on the *S* locus, including *S-RNase* and *SLF* genes, the key regions/genes that regulate the reproduction system of *Fragaria.* Here, we identified 33 members of *SLF*/*SLF-like F-box* genes from the *F. vesca* strawberry genome, 12 of which were clustered in Clade S and located within one end of the *S* locus of *F. vesca* in tandem duplications (Fvb6: 3,860–4,140k) (fig. 4*b* and *c*). This 280-kb region contains three and five 10-kb windows with evidence of selective sweep in *F. vesca* and *F. nilgerrensis*, respectively (fig. 4*c*). In particular, four *FvSLFs* were located in two selective sweeps (Fvb6: 3,960–3,970k; Fvb6: 4,110–4,120k) of *F. nilgerrensis*, while the latter window (containing one *FvSLF*) was also identified in selective sweeps of *F. vesca* (fig. 4*c*). Meanwhile, we screened six *S-RNase*/*S-RNase-like* genes in *F. vesca*, two of which were clustered in Class III and located at the other end of the *S* locus (Fvb6: 4,630–4,651k; supplementary fig. S10, Supplementary Material online), approximately 500 kb away from the region of tandem duplicated *SLFs*. However, no *S-RNase*/*S-RNase-like* genes overlapped with either of the selective sweeps for all wild diploid strawberries (supplementary table S13, Supplementary Material online). These findings indicated that the *FvSLFs* may contribute potential roles in the mating system transitions in wild diploid strawberries.

Furthermore, these two windows fall in high introgression regions even with the top 1% highest introgression index (fig. 4*c* and supplementary table S7, Supplementary Material online). We also detected introgression signals between *F. mandshurica*/*F. viridis* (*P3*) and *F. nipponica*/*F. pentaphylla*/*F. nubicola* (*P2*) at the window Chr6: 3,960–3,970k, and signals between *F. viridis* (*P3*) and *F. chinensis*/*F. nipponica*/*F. pentaphylla*/*F. nubicola* (*P2*) at the window Chr6: 4,110–4,120k (fig. 4*d* and supplementary table S6, Supplementary Material online), but none was found between two SC species for either of these two windows. Combining with evidence of ancient introgression by *D*_FOIL_ and PhyloNet (fig. 3*b* and *c*), we hypothesize that these two windows experienced ancient introgression from *F. viridis* (and/or *F. mandshurica*) to the ancestor of the lineage of *F. chinensis*, *F. nipponica*, *F. nubicola*, and *F. pentaphylla*, which may directly contribute to the mating system transition from SC (the ancestor of the lineage of *F. chinensis*, *F. nipponica*, *F. nubicola*, *F. pentaphylla*, *F. nilgerrensis*, *F. daltoniana*, and *F. iinumae*) to SI (the ancestor of the lineage of *F. chinensis*, *F. nipponica*, *F. nubicola*, and *F. pentaphylla*) (fig. 4*d*).

## Discussion

### Recombination-aware Phylogenomics of the Wild Diploid Strawberries

The phylogenetic relationships among diploid *Fragaria* species have not been resolved even in large multilocus datasets (Edger et al. 2019; Liston et al. 2020; Feng et al. 2021; Qiao et al. 2021; Fan et al. 2022). Previous phylogenomic studies based on genome-wide loci demonstrated conflicting topological positions for many diploid species (supplementary fig. S1, Supplementary Material online). Although these discordances can result from artifacts arising from tree inference methods, both gene flow and ILS accompanying rapid radiation or recently diverged lineages can overwhelm the genealogical signal of the original population branching pattern, and lead to gene trees that contradict the true species tree (Shen et al. 2017; Irisarri et al. 2018; Li et al. 2019). However, despite the rapid development of more sophisticated approaches (Edwards 2009; Edwards et al. 2016; Mirarab et al. 2016; Xu and Yang 2016), estimating species tree that explicitly account for gene flow and ILS remains challenging for lineages with an extensive history of hybridization and introgression.

In the present study, by partitioning genomic windows into different levels of recombination, we show that topology weightings are significantly correlated with the recombination rate (fig. 2*d* and *e*). Specifically, we found that the genomic windows with low recombination exhibited lower levels of introgression and ILS compared to the medium to high recombination regions (fig. 3*d* and supplementary fig. S4, Supplementary Material online). As a result, the low recombination regions tend to harbor a greater proportion of topologies reflecting speciation and, therefore, are more likely to give a topology matching the species tree (Topo2 in fig. 2*a*). These results are consistent with previous reports of enriched true species trees in genomic regions with low and no recombination (Pease and Hahn 2013; Fontaine et al. 2015; Edelman et al. 2019; Li et al. 2019; Martin et al. 2019; Hennelly et al. 2021; Owens et al. 2022). For example, Pease and Hahn (2013) found genomic regions with low or no recombination showed significantly stronger support for the putative species tree in the presence of ILS, because low recombination regions have lower effective population size (*Ne*) and, therefore, less retention of shared ancestral polymorphisms than higher-recombination regions. Recent phylogenomic studies of *Heliconius* butterflies (Edelman et al. 2019; Martin et al. 2019), the cat family Felidae (Li et al. 2019), grey wolves (Hennelly et al. 2021), and sunflowers (Owens et al. 2022) showed that the most likely species tree is notably enriched within low recombination regions of sex chromosomes in the presence of gene flow, owing to the strong linkage in low recombination regions which leads to the more effective removal of deleterious alleles introduced through hybridization (Nachman and Payseur 2012; Schumer et al. 2018). Together, these findings indicate that phylogenomic parameters inferred from whole genome data may be misleading and highlight the consideration of taking into account recombination rate variation in phylogenetic reconstruction, especially for lineages that experienced extensive hybridization.

### Genomic Landscape of Introgression Across Wild Diploid *Fragaria* Species

Genomic analyses have proved mounting evidence that introgression seems to be more common than previously thought, and that mosaic genomes are more common than homogeneous genomes (Taylor and Larson 2019). However, the genomic proportion and landscape of introgression are not well understood. Most previous studies are focused on the hybridization and gene flow between single or few number pairs of taxa, which makes it hard to infer general patterns at a broad scale. In this study, we applied *D*-statistic and *f*_hom_ values to detect and quantify introgression among ten wild diploid strawberries and observed that 0.2% to 16.4% of the genomes were introgressed ancestry (fig. 3*a* and supplementary table S5, Supplementary Material online). The genomic proportion of introgression in *Fragaria* is similar to 2.0–8.0% among six North American wild grapes (Morales-Cruz et al. 2021) and 0.4–10.7% among the seven species from section *Populus* of the genus *Populus* (Liu et al. 2022), but higher than that reported for wild tomato species (0.2–2.5%; Hamlin et al. 2020). However, it is noteworthy that ancient introgression events can affect the estimation of genomic introgression among extant species (Malinsky et al. 2018). Therefore, our detected high genomic proportion of introgression in *Fragaria* might be overestimated due to ancient introgression and should be interpreted with caution. Nevertheless, the similar levels of genomic introgression observed in *Fragaria* and other species suggest that introgression shapes substantial genomic variation of wild plant genomes.

The fate of any given introgressed allele will depend not only on the adaptive value of its associated variants but also on the local genomic landscape in terms of recombination rates and the nature of linked genes (Martin and Jiggins 2017). In this study, we found that genomic windows with high introgression tend to be retained in the regions of high recombination rate, whereas low introgression regions are concentrated in genomic regions with low recombination rates (fig. 3*d*). Such a strong positive correlation between introgression and recombination rate has been observed in genomic studies of a range of taxa, including butterfly (Martin et al. 2019), alpine bumblebees (Christmas et al. 2021), maize (Calfee et al. 2021), wild grapes (Morales-Cruz et al. 2021), *Populus* species (Liu et al. 2022), and sunflowers (Owens et al. 2022). These findings are consistent with theoretical expectations that introgressed genomic alleles (mostly deleterious mutations) would break down rapidly during subsequently repeated backcrossing following initial hybridization and tend to be retained due to the selection against deleterious foreign alleles within high recombination regions (Schumer et al. 2018; Martin et al. 2019; Morales-Cruz et al. 2021). Given the fact that gene regions have relatively low recombination rate (Martin et al. 2019), it is not unexpected for the observed negative relationship between gene density and introgressed ancestry (fig. 3*d*). However, it is difficult to test whether such regions directly shape barriers to introgression (Martin et al. 2019). Nevertheless, we found a stronger correlation between introgression and gene density, than introgression and recombination rate (fig. 3*d*), which might support the direct contribution of gene density on the genomic landscape, because gene-rich foreign regions contain numerous deleterious mutations, and thus are less likely to be introgressed (Morales-Cruz et al. 2021). However, introgression of large genomic regions is known to occur (Dasmahapatra et al. 2012). Furthermore, we also found extremely low levels of recombination rate in high genetic differentiation (*F*_ST_) regions, in addition to a negative relationship between *F*_ST_ and recombination rate (fig. 3*d*), which supports a role for linked selection (Burri 2017). Overall, our findings demonstrate that recombination rate variation is an important factor in shaping the landscape of genomic introgression and differentiation among wild diploid *Fragaria* species.

### Selective Sweep of *SLFs* May Contribute to Mating System Transitions

SI is a widespread genetic system in angiosperms and occurs in approximately 39% of plant species (Igic et al. 2008). SI systems fall into two major classes: gametophytic self-incompatibility (GSI) and sporophytic self-incompatibility (SSI) (Hiscock and McInnis 2003). GSI is quite common in families such as Rosaceae, Solanaceae, and Plantaginaceae, and SI is regarded as the ancestral state (Igic and Kohn 2001). The *Fragaria* genus provides an exceptional model to study mating system transitions, not only because it underwent a full range of sexual systems from hermaphroditism (diploid) to dioecy that is associated with all increases in ploidy in the genus, and back again from subdioecy of a wild octoploid to hermaphroditism of commercial strawberry along domestication, but also because of variation in SI/SC of diploid species (Liston et al. 2014). Combining the results of model comparisons and ancestral mating system reconstruction, we propose the possible transition from SC to SI along the ancestor of the lineage of *F. chinensis*, *F. nipponica*, *F. nubicola*, and *F. pentaphylla* (fig. 4*d*). However, Chen et al. (2022) considered that wild diploid *Fragaria* evolved multiple losses of SI, according to a phylogeny inferred by orthologous single-copy genes. Although the loss of SI is believed to be more frequently than the transition from SC to SI (Igic et al. 2008), bidirectional transitions were inferred in the Asteraceae and the Brassicaceae (Ferrer and Good-Avila 2007; Sherman-Broyles and Nasrallah 2008). In particular, Njuguna et al. (2013) also proposed that both loss and gain of SI are possible in *Fragaria* according to phylogeny estimated from the chloroplast genome.

Introgression from closely related species can introduce adaptive genetic variation that may affect traits. However, linking adaptive traits with signatures of introgression is still rare and remains a challenge in most systems (Taylor and Larson 2019; Morales-Cruz et al. 2021). As well known, the GSI system contains the female *S* determinant (*S-RNase*) in the pistil, and the male *S* determinant (*SLF* or called *SFB*, *SFBB*, *SLFL*) in pollen, while the *SLF* genes were usually derived from tandem duplications (Akagi et al. 2016). The deletion/mutation of specific *SLF* in SC haplotypes could be responsible for the loss of SI in the tribe Maleae and Amygdaleae (Ushijima et al. 2004; Sonneveld et al. 2005; Kakui et al. 2011; Ashkani and Rees 2016). However, the knowledge about the SI/SC mechanism for *Fragaria* is only limited to the identification of *S*-*RNase* genes (Bošković et al. 2010; Du et al. 2021; Chen et al. 2022). Here, we identified a roughly 280 kb region in the *S*-locus of *F. vesca* (Fvb6: 3,860–4,140k), which contained 27 genes including 12 *FvSLFs* and several windows under selective sweep (fig. 4*b* and *c*), and may potentially play a role in mating system transitions. More interesting, the two selective sweep regions (Fvb6: 3,960–3,970k; Fvb6: 4,110–4,120k) that contain four *FvSLFs* exhibited ancient introgression from *F. viridis* (and/or *F. mandshurica*) to the ancestor of the lineage of *F. chinensis*, *F. nipponica*, *F. nubicola*, and *F. pentaphylla*, which could further explain the regain of SI in this lineage, following the initial transitions from SI to SC (figs. 3*b,*3*c*, and 4*d* and supplementary table S7, Supplementary Material online). Our finding on the mechanism of mating system transitions is important for genetic breeding and germplasm innovation in strawberries, and also provides a novel example of adaptive introgression related to mating system transitions in plants.

## Materials and Methods

### Plant Material and Genome Resequencing

We collected 64 samples from ten wild diploid *Fragaria* species in the China National Germplasm Repository for Strawberry (Nanjing) at the Jiangsu Academy of Agricultural Sciences (Nanjing, China), with detailed sampling information provided in supplementary table S1, Supplementary Material online. Genomic DNA was extracted from fresh leaves with the plant DNA extraction kit (AU31111, Bioteke, China). Libraries were constructed with a short insert size of 350 for each sample and then subjected to sequence (PE150) on Illumina NovaSeq 6000 platform.

### SNP Calling and Genotyping

We downloaded genome survey data of *F. viridis* (designated as Vir-0), *F. nubicola* (designated as Nub-0), *F. nilgerrensis* (designated as Nil-0), *F. iinumae* (designated as Iin-0), and *Potentilla microphylla* (designated as Pmic) from SRA database under SRR11833746, SRR11833747, SRR11833748, SRR9217951, and PRJEB18433, respectively. The first three datasets were generated from our previous study (Feng et al. 2021). After filtering low-quality reads of these five samples, plus 64 newly sequenced wild strawberries, according to pipeline QC_pe (Feng et al. 2017), we mapped the clean data of each individual to the *F. vesca* Genome v4.0 (https://www.rosaceae.org/species/fragaria_vesca/genome_v4.0.a1; Edger et al. 2017) by BWA (Li and Durbin 2009) and then conducted SNP calling using SAMtools v0.1.19 (Li et al. 2009), Picard tool v1.119 (http://broadinstitute.github.io/picard/), and GATK v4.1.4.0 (DePristo et al. 2011). Only sites with a quality score above 30 were kept in HaplotypeCaller. Furthermore, we joined genotype files (gVCF) of all individuals together and filtered the variant sites using GATK VariantFiltration with the expression “QD < 2.0 || FS > 60.0 || MQ < 40.0 || MQRankSum < -12.5 || ReadPosRankSum < −8.0.” Additionally, we removed the indels and kept the sites for downstream analyses, according to minQ 30, max-missing 0.67, max-alleles 2, max-meanDP 500, min-meanDP 10.

Finally, we retained 69 genotypes (including the outgroup Pmic), and 14,175,029 high-quality SNPs, plus 68,550,711 invariant sites (Signed as Dataset 1). Meanwhile, we excluded minor allele frequency sites (maf 0.05) by VCFtools (Danecek et al. 2011) to generate Dataset 2 that contains 5,751,545 SNPs and 69 genotypes. Additionally, we excluded genotypes of outgroup Pmic and removed highly correlated SNPs by PLINK (Purcell et al. 2007) with the parameter indep-pairwise of 50 5 0.2, yielding Dataset 3 (459,031 unlinked SNPs, 68 genotypes).

### Population Structure Analyses

To investigate population structure in *Fragaria*, we performed admixture analyses according to Dataset 3, by using STRUCTURE (Pritchard et al. 2000) and PCA. Firstly, we ran eight independent STRUCTURE with different *K* values ranging from 2 to 11 hypothetical ancestral populations, setting the length of burn-in and number of MCMC replications after burn-in as 20,000 and 100,000, respectively. Then, we calculated the best *K* value by the Δ*K* method using STRUCTURE HARVESTER (Earl and vonHoldt 2012) and averaged the cluster assignment from eight replications by CLUMPP (Jakobsson and Rosenberg 2007). Also, we conducted PCA for 68 individuals from the 10 diploid strawberry species with GCTA (Yang et al. 2011) and displayed the result between PC1 and PC2/PC3.

### Recombination Rate and Gene Density

To estimate the recombination rate along the strawberry genome, we calculated the population-scaled recombination rate for each 10 kb nonoverlapping sliding window across the ten wild diploid strawberries. We phased each chromosome according to unlinked SNPs of 68 *Fragaria* individuals (Dataset 3) by using Beagle (Browning and Browning 2009). Then, we used FastEPRR (Gao et al. 2016) to estimate the recombination rate (Rho) per 10 kb windows by combining all the species together. FastEPRR is a widely used R package for the rapid and accurate estimation of population recombination rates from DNA polymorphisms. To investigate the gene density across seven strawberry chromosomes, we calculated the proportion of gene regions at each 10 kb nonoverlapping sliding window based on gff files of *Fragaria vesca* v4.0.a1.

### Phylogenetic Tree Estimation

We applied four approaches to reconstruct phylogenetic relationships among wild diploid *Fragaria* species. Firstly, we divided the genome (Dataset 1) into 10 kb nonoverlapping sliding windows but only focused on the windows with at least 200 sites and 20 parsimony-informative sites (PIS), and then constructed ML trees for each window by using IQ-TREE (Nguyen et al. 2015). Furthermore, we also removed the trees with the average bootstrap support value less than 60%, resulting in 19,295 ML trees/windows. We used a coalescent-based summary method (ASTRAL; Mirarab and Warnow 2015) to construct the species tree based on 19,295 ML trees (Approach 1). Meanwhile, we adopted a concatenation approach to infer ML trees by using IQ-TREE (Nguyen et al. 2015) according to concatenation nucleotide sites from 19,295 windows (Approach 2). Additionally, we rebuilt ASTRAL trees and ML trees at chromosomal scales (Fvb1–Fvb7), and for five groups that were equally classified by recombination rate of corresponding windows (low, medium–low, medium, medium–high, and high recombination), respectively. In addition, we inferred divergence times and substitution rates for 68 accessions representing the 10 wild diploid strawberries by using r8s (Sanderson 2003) with parameter “smooth” of 0.01, according to ML tree reconstructed with low recombination regions, setting a secondary age calibration, the crown age of genus *Fragaria* at 6.37 Ma (Feng et al. 2021).

The third approach is that we used a site-based coalescent method (SVDQuartets; Chifman and Kubatko 2014) to infer species tree according to Dataset 2. Lastly and the fourth approach, we assembled the chloroplast genome for each of 69 individual by NOVOplasty (Dierckxsens et al. 2017) and built the chloroplast ML tree by using MAFFT (Katoh and Standley 2013) and IQ-TREE (Nguyen et al. 2015).

### Topology Weighting

To explore evolutionary relationships among wild diploid strawberries, we carried out *Twisst* (Martin and Van Belleghem 2017) to quantify topology weighting among and within chromosomes. First, we generated 19,295 species trees from each ML tree of 10 kb nonoverlapping sliding windows by ASTRAL (Mirarab and Warnow 2015) under the map of the relations between individuals and species, setting *P. microphylla* as the outgroup. The software *Twisst* does not scale to more than eight taxa combinations; therefore, we combined monophyletic clade into one taxa combination (e.g., group A contains *F. chinensis* and *F. nipponica*, group D contains *F. nilgerrensis* and *F. daltoniana*, group G contains *F.vesca* and *F. mandshurica*), according to the variations of *Fragaria* phylogeny in previous and this study (supplementary figs. S1 and S2, Supplementary Material online). For eight taxa combinations, there is a maximum of 10,395 possible rooted, bifurcating tree topologies. Here, we applied *Twisst* (Martin and Van Belleghem 2017) to classify 19,295 species trees into 3,441 possible topologies, yielding weightings for each topology. Moreover, we focused on the main topologies with occurrence frequency of over 1.5%, and drew the distribution of the four main topologies along chromosomes. Lastly, we analyzed average weightings for the main possible topologies at chromosomal scales and groups with partitioned recombination rates.

### Admixture and Introgression Analyses

To evaluate the contribution of hybridization and ILS on the discordance of topology across the genome, we performed *MSCquartets* analyses (Rhodes et al. 2021), estimated quartet score (Mirarab and Warnow 2015), and detected the signals for introgression (Solis-Lemus et al. 2017; Wen et al. 2018). Firstly, we carried out quartetTreeTest with the “T3 model” in the *MSCquartets* package (Rhodes et al. 2021) based on the 19,295 species trees inferred from 10 kb nonoverlapping sliding windows (same as the input file of topology analyses), with the rejection level of 1e^−6^. The package would generate a series of plots for all four-taxon subsets with a red triangle (reject tree) or blue circle (fail to reject tree). More blue circles lying closer proximity to the centroid represent substantial ILS, while closer to the vertex represent little ILS. Secondly, we used “q option” of ASTRAL (Mirarab and Warnow 2015) to calculate the normalized quartet score (i.e., the proportion of quartet trees in the input trees that are satisfied by the ASTRAL tree) at the whole genome (19,295 species trees), chromosomal levels (ranging from 2,110 to 3,590 trees), and groups with partitioned recombination rate (3,859 trees for each group). The score is a number between zero and one, the lower this number indicates the more discordant the input trees. Thirdly, we performed *D*_FOIL_ analyses (Pease and Hahn 2015) to detect introgression based on a five-taxon phylogeny (((*P1*, *P2*), (*P3*, *P4*)), *O*), where *O* is the outgroup (*Pmic*) and *P1* to *P4* are the ingroups, and the divergence between *P3* and *P4* should be earlier than that between *P1* and *P2*. *D*_FOIL_ analysis could distinguish the ancient introgression and post-speciation gene flow, and also estimate the direction of post-speciation gene flow. Here, we applied the software *D*_FOIL_ to examine the introgression signals in 55,207 individual combinations from 34 species combinations, according to the species tree inferred from low recombination regions. Fourthly, we adopted the Infer_Network_MPL model (Wen et al. 2018) to detect gene flow, according to 19,295 rooted species trees, by setting maximum reticulations of 5. Meanwhile, we applied SNPs2CF (https://github.com/melisaolave/SNPs2CF/) to compute concordance factors from unlinked SNPs of 19 represented individuals (Chi-3, Chi-7, Nip-1, Nub-0, Nub-1, Pen-3, Pen-7, Nil-0, Nil-4, Dal-1, Dal-2, Iin-0, Iin-1, Vir-0, Vir-9, Ves-1, Ves-11, Man-2, and Man-3), which could greatly reduce the computational complexity. Then, we compared and estimated the hypothetical hybridization with hman setting from 0 to 10, by using PhyloNetworks (Solis-Lemus et al. 2017).

To further detect and characterize introgression across the *Fragaria* genome, we used Dsuite (Malinsky et al. 2021) and *ABBABABAwindows*.*py* script (Martin et al. 2015). Firstly, we applied the *D*_min_ model of program Dtrios in Dsuite (Malinsky et al. 2021) to perform the ABBA-BABA test and calculate the overall *D* statistic and associate *P* value for 120 (.) four-taxa trios, 95 trios of which had adjusted *P* value < 0.01, and were defined as trios with significant introgression signals. Furthermore, we also followed the formula, *f*_hom_ = S (*P1*, *P2*, *P3*, *O*)/S (*P1*, *P3*, *P3*, *O*) = (*ABBA1*-*BABA1*)/(*ABBA2*-*BABA2*) (Martin et al. 2015; Pulido-Santacruz et al. 2020), to calculate *f*_hom_ value for 95 trios with significant introgression signals. Then, we counted the *f*_dM_ values in each 10 kb nonoverlapping sliding window for each of the 95 trios by using *ABBABABAwindows*.*py* script (Martin et al. 2015), removing the windows containing less than 10 SNPs. We defined putatively introgression regions (pIR) as windows with the highest *x*% of *f*_dM_ values, where *x* was determined for each trio by the *f*_hom_ estimate, and then calculated an introgression index for each window as the proportion of trio with pIRs. The windows that have the top approximately 5% highest introgression index were defined as high introgression regions (the threshold value is 0.1578 in this study), while the windows having an introgression index equal to 0 were defined as low introgression regions.

Similarly, we used VCFtools (Danecek et al. 2011) to calculate the genetic differentiation (*F*_ST_) value for each 10 kb nonoverlapping sliding window of 21 (.) combinations that were pairwise of the seven wild species containing at least three individuals (i.e., *F. chinensis*, *F. nubicola*, *F. pentaphylla*, *F. nilgerrensis*, *F. viridi*, *F. vesca*, and *F. mandshurica*). For each combination, we marked the window with the top 5% of *F*_ST_ value as the *F*_ST_ outlier and defined the *F*_ST_ index of each window as the proportion of the combination with the *F*_ST_ outlier. We then ranked the windows by *F*_ST_ index and classified the windows with the highest approximately 5% *F*_ST_ index (>0.3333) as high *F*_ST_ regions (i.e., introgression barrier regions). Lastly, we analyzed the average percentage of windows with high introgression and low introgression/introgression barrier regions, binned according to their recombination rate and gene density.

### Detection of Selective Sweeps

We used a combination of a haplotype-based method (selscan; Szpiech and Hernandez 2014) and a composite evaluation approach (RAiSD; Alachiotis and Pavlidis 2018) to screen the regions under positive selection for the seven *Fragaria* species containing at least three individuals (like *F*_ST_ analysis), following our previous pipeline (Hu et al. 2022). Firstly, we calculated raw XP-nSL scores for each chromosome on all 42 comparisons of seven species by using selscan v1.3.0 (Szpiech and Hernandez 2014) and then normalized the XP-nSL value across 10 kb nonoverlapping windows using norm v1.3.0 (Szpiech and Hernandez 2014). For each species, we collected the windows with the top 1% extreme score, setting either one of six other species as a reference, respectively. After removing redundancy, the remaining windows were used for downstream analysis. Secondly, we applied RAiSD (Alachiotis and Pavlidis 2018) to generate raw μ value across the genome for each of the seven species and then calculated the average μ value for 10 kb nonoverlapping windows. Here, we defined the windows with the highest 5% values as candidate regions. Finally, the overlap regions from the above two approaches were regarded as the highly confident selective sweep regions for each wild diploid strawberry. We compared the shared and specific windows under selective sweep regions of the seven *Fragaria* species and drew the Upset diagram by TBtools (Chen et al. 2020).

### GO Enrichment

We applied the topGO script (http://bioconductor.uib.no/2.7/bioc/vignettes/topGO/), following our previous pipeline (Feng et al. 2020), to analyze the gene ontology enrichment of genes in the high introgression and low introgression/introgression barrier regions across the wild diploid strawberries, as well as genes from the selective sweep regions of the seven *Fragaria* species, setting all *F. vesca* genes as background. Only the lowest-level GO terms in MF and BP, with a *P* value <0.05, were retained as enriched terms, whereas the *P* value was calculated according to a “classic” algorithm under Fisher's test.

### Identification of *SLFs* and *S-RNases* in *F. vesca* Genome

Following the pipeline of Akagi et al. (2016), we scanned *F-box* genes in *F. vesca* genome (v4.0) by using BLASTP against *SFB* gene (AB111521) or *SLF2* gene (AB280954) from *Prunus avium* with the cutoff of 1e^−19^ and further removed the genes that were absent of the F-box domain (PF00646.33) by Pfam search (Mistry and Finn 2007). Furthermore, we used IQ-TREE (Nguyen et al. 2015) to construct the ML tree of *SLF*/*SLF-like* F-box genes from the strawberry genome and partial F-box genes from peach (prefix of “ppa’), cherry, etc., based on CDS sequences guided by the alignment of protein sequences by using the PAL2NAL script (Suyama et al. 2006). Finally, we classified these *F-box* genes into Clades A, B, S, and Others, according to the standard of Akagi et al. (2016), whereas the genes in Clade S were considered as *SLFs* that contribute to pollen *S* function. Similarly, we followed the pipeline of Morimoto et al. (2015) to identify *S-RNase-like* genes according to *S_3_-RNase* gene (AB010306) from *P. avium* and ribonuclease T2 Pfam domain (PF00445.18). According to the standard of Morimoto et al. (2015), the members in Class III were regarded as candidate *S-RNases* which may have pistil *S* function. The region containing at least a single pistil *S* determinant *S-RNase* and several pollen *S-*related *SLFs* in tandem duplications was defined as the *S* locus in *F. vesca*.

### Estimation of Ancestral Mating System Type in Wild Diploid Strawberries

We simulated the probability of ancestral mating system states at each ancestral node using BayesTraits implemented in RASP 4 (Yu et al. 2020), according to phylogenetic relationship at low recombination regions with divergence time, and current mating systems (SI or SC) of the ten wild diploid strawberries described by Njuguna et al. (2013). With the *ape* package in phytools (Revell 2012), two models (symmetric and asymmetric transition rates) in ML analyses were compared to determine whether the gain rate (transition rate from SC to SI) is significantly different from the loss rate (transition rate from SI to SC).

## Supplementary Material

msad049_Supplementary_Data

## Data Availability

The genome resequencing data has been deposited to the SRA database at NCBI under BioProject PRJNA879993.
