# Supplementary material for: Recombination Variation Shapes Phylogeny and Introgression in Wild Diploid Strawberries
Source: Mol Biol Evol. 2023 Mar 2;40(3):msad049. doi: 10.1093/molbev/msad049 (PMC10015625; doi:10.1093/molbev/msad049)
Supplement: msad049_Supplementary_Data [file msad049_supplementary_data.zip › Figure S2_Summary of phylogenetic relationships of wild diploid strawberries.pdf]

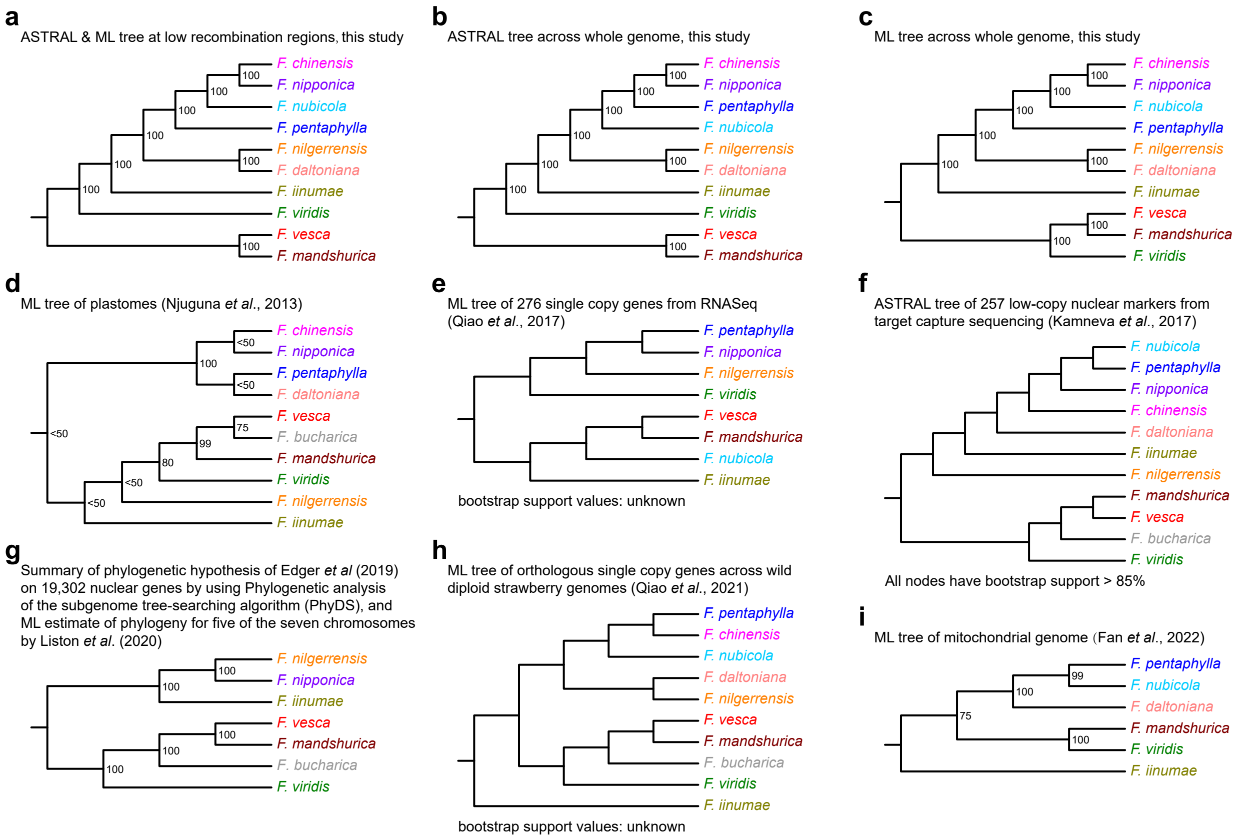
a
ASTRAL & ML tree at low recombination regions, this study
F. chinensis
F. nipponica
F. nubicola
F. pentaphylla
F. nilgerrensis
F. daltoniana
F. iinumae
F. viridis
F. vesca
F. mandshurica
b
ASTRAL tree across whole genome, this study
F. chinensis
F. nipponica
F. pentaphylla
F. nubicola
F. nilgerrensis
F. daltoniana
F. iinumae
F. viridis
F. vesca
F. mandshurica
c
ML tree across whole genome, this study
F. chinensis
F. nipponica
F. nubicola
F. pentaphylla
F. nilgerrensis
F. daltoniana
F. iinumae
F. vesca
F. mandshurica
F. viridis
d
ML tree of plastomes (Njuguna et al., 2013)
F. chinensis
F. nipponica
F. pentaphylla
F. daltoniana
F. vesca
F. bucharica
F. mandshurica
F. viridis
F. nilgerrensis
F. iinumae
e
ML tree of 276 single copy genes from RNASeq (Qiao et al., 2017)
F. pentaphylla
F. nipponica
F. nilgerrensis
F. viridis
F. vesca
F. mandshurica
F. nubicola
F. iinumae
bootstrap support values: unknown
f
ASTRAL tree of 257 low-copy nuclear markers from target capture sequencing (Kamneva et al., 2017)
F. nubicola
F. pentaphylla
F. nipponica
F. chinensis
F. daltoniana
F. iinumae
F. nilgerrensis
F. mandshurica
F. vesca
F. bucharica
F. viridis
All nodes have bootstrap support > 85%
g
Summary of phylogenetic hypothesis of Edger et al (2019) on 19,302 nuclear genes by using Phylogenetic analysis of the subgenome tree-searching algorithm (PhyDS), and ML estimate of phylogeny for five of the seven chromosomes by Liston et al. (2020)
F. nilgerrensis
F. nipponica
F. iinumae
F. vesca
F. mandshurica
F. bucharica
F. viridis
h
ML tree of orthologous single copy genes across wild diploid strawberry genomes (Qiao et al., 2021)
F. pentaphylla
F. chinensis
F. nubicola
F. daltoniana
F. nilgerrensis
F. vesca
F. mandshurica
F. bucharica
F. viridis
F. iinumae
bootstrap support values: unknown
i
ML tree of mitochondrial genome (Fan et al., 2022)
F. pentaphylla
F. nubicola
F. daltoniana
F. mandshurica
F. viridis
F. iinumae
