# Supplementary figures and images for: Recombination Variation Shapes Phylogeny and Introgression in Wild Diploid Strawberries
Source: Mol Biol Evol. 2023 Mar 2;40(3):msad049. doi: 10.1093/molbev/msad049 (PMC10015625; doi:10.1093/molbev/msad049)

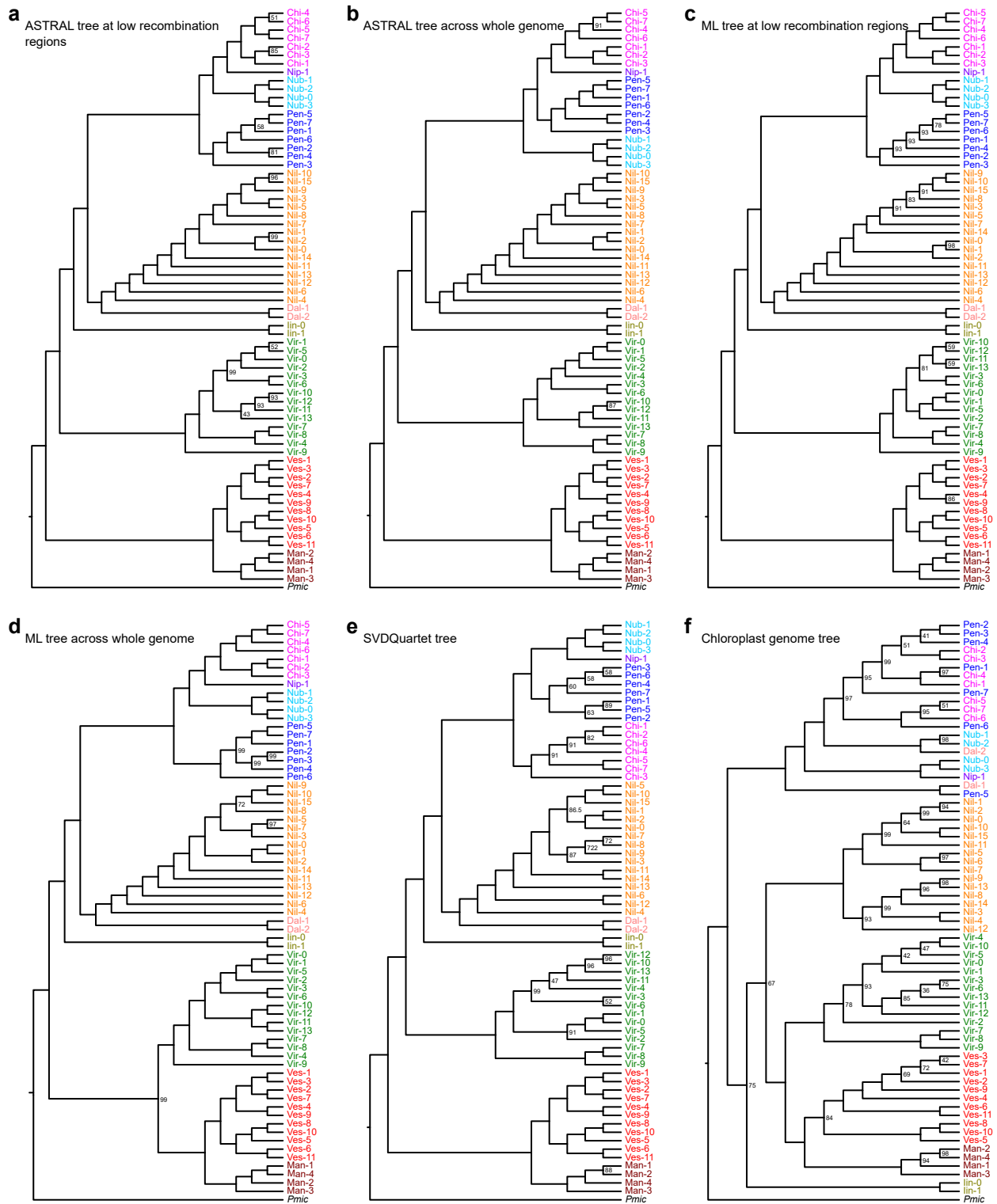

Supplement: msad049_Supplementary_Data [file msad049_supplementary_data.zip › Figure S1_Phylogenetic relationship of the ten wild diploid strawberries.pdf]

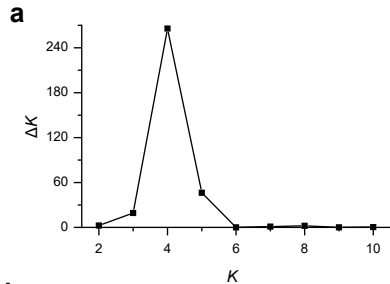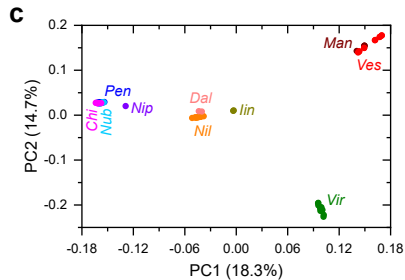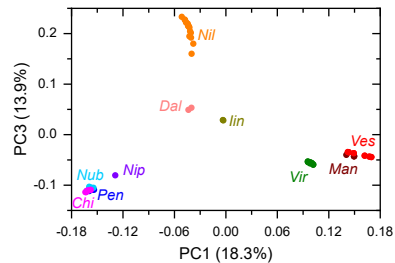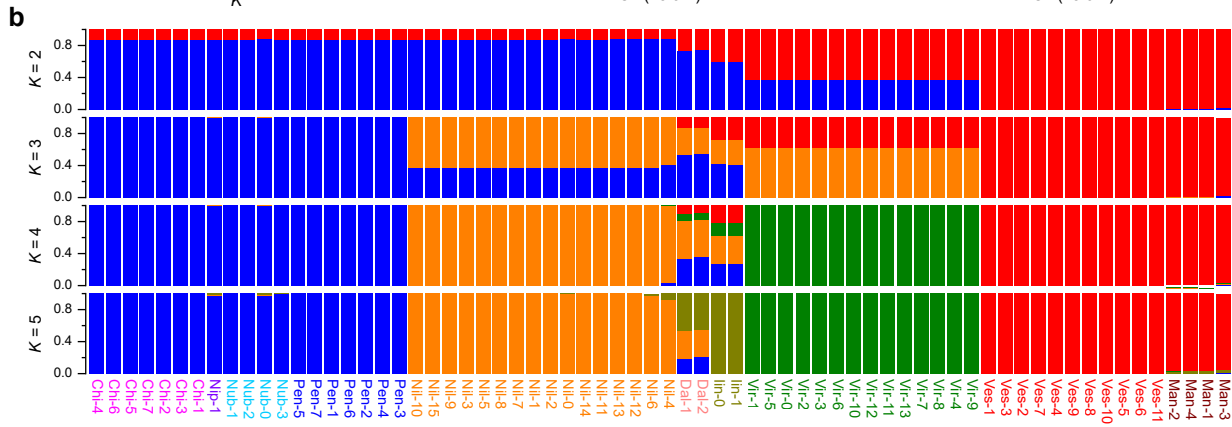

Supplement: msad049_Supplementary_Data [file msad049_supplementary_data.zip › Figure S3_Structure & PCA.pdf]

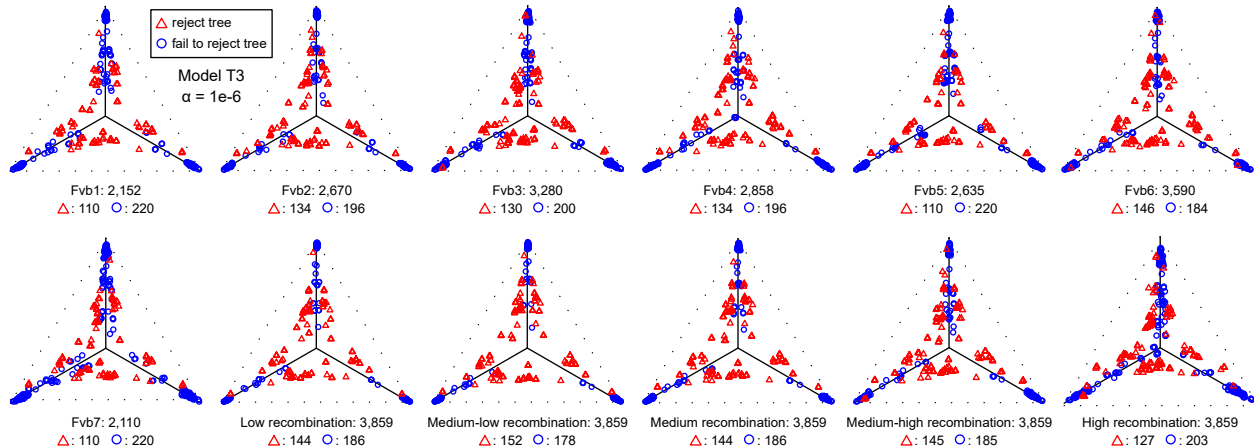

Supplement: msad049_Supplementary_Data [file msad049_supplementary_data.zip › Figure S4_Simplex plots of quartet concordance factors.pdf]

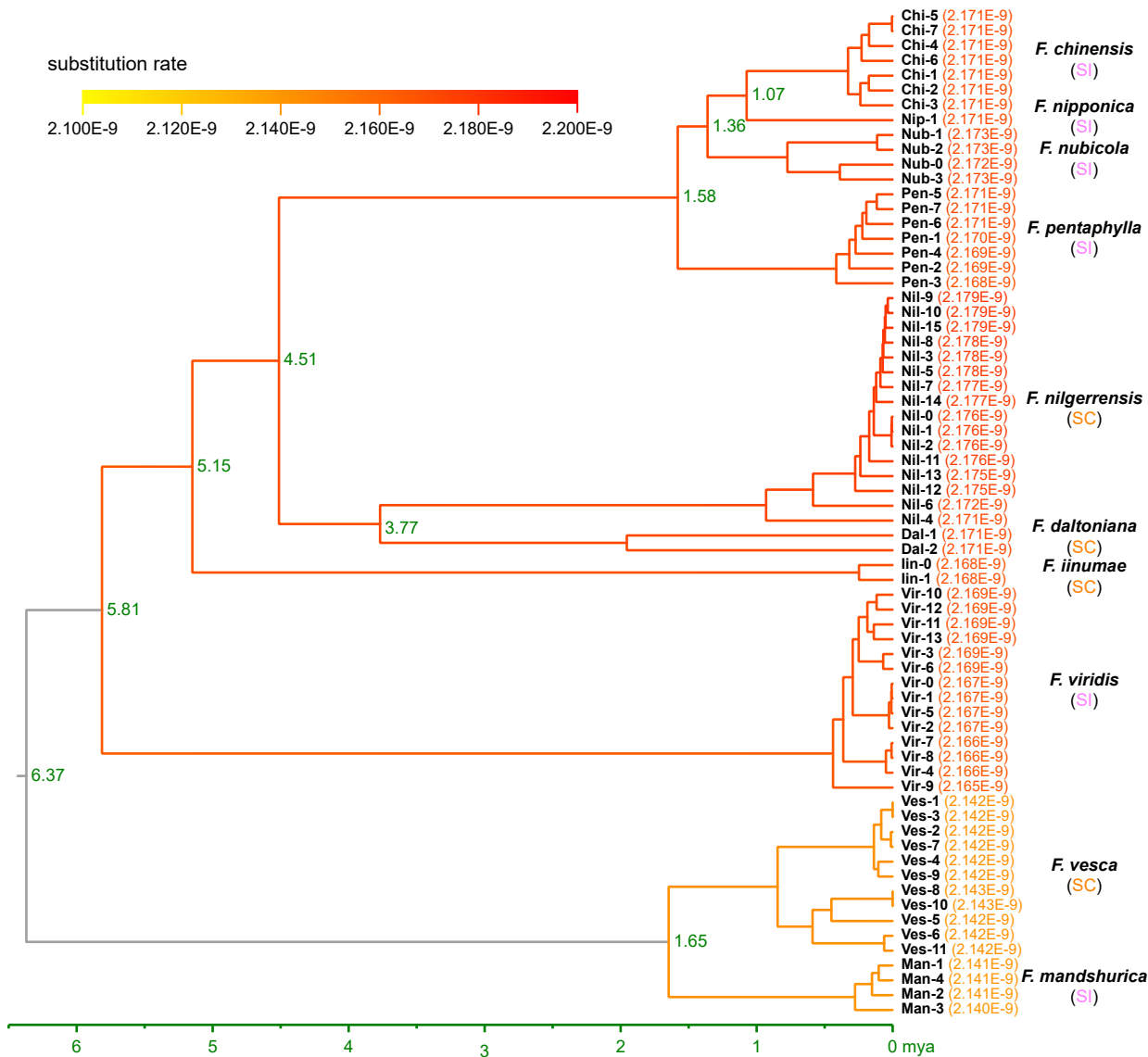

Supplement: msad049_Supplementary_Data [file msad049_supplementary_data.zip › Figure S5_Divergence time and substitution rate for 68 individuals.pdf]

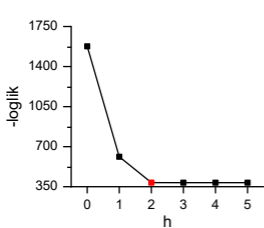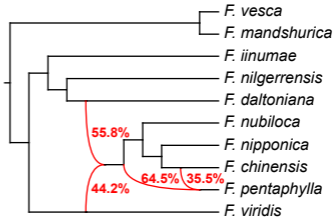

Supplement: msad049_Supplementary_Data [file msad049_supplementary_data.zip › Figure S6_PhyloNetworks.pdf]

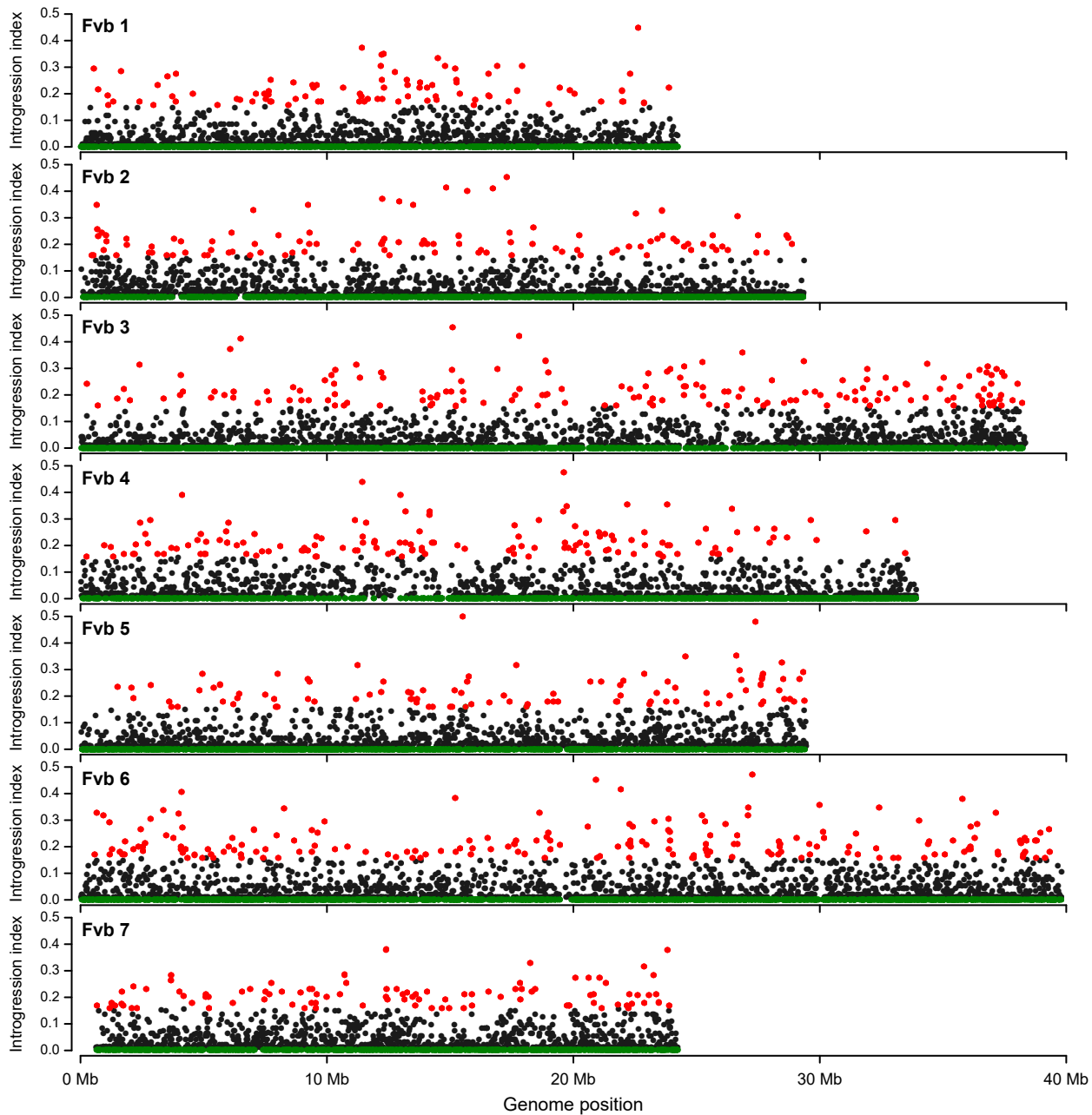

Supplement: msad049_Supplementary_Data [file msad049_supplementary_data.zip › Figure S7_Genome distribution of high and low introgression regions.pdf]

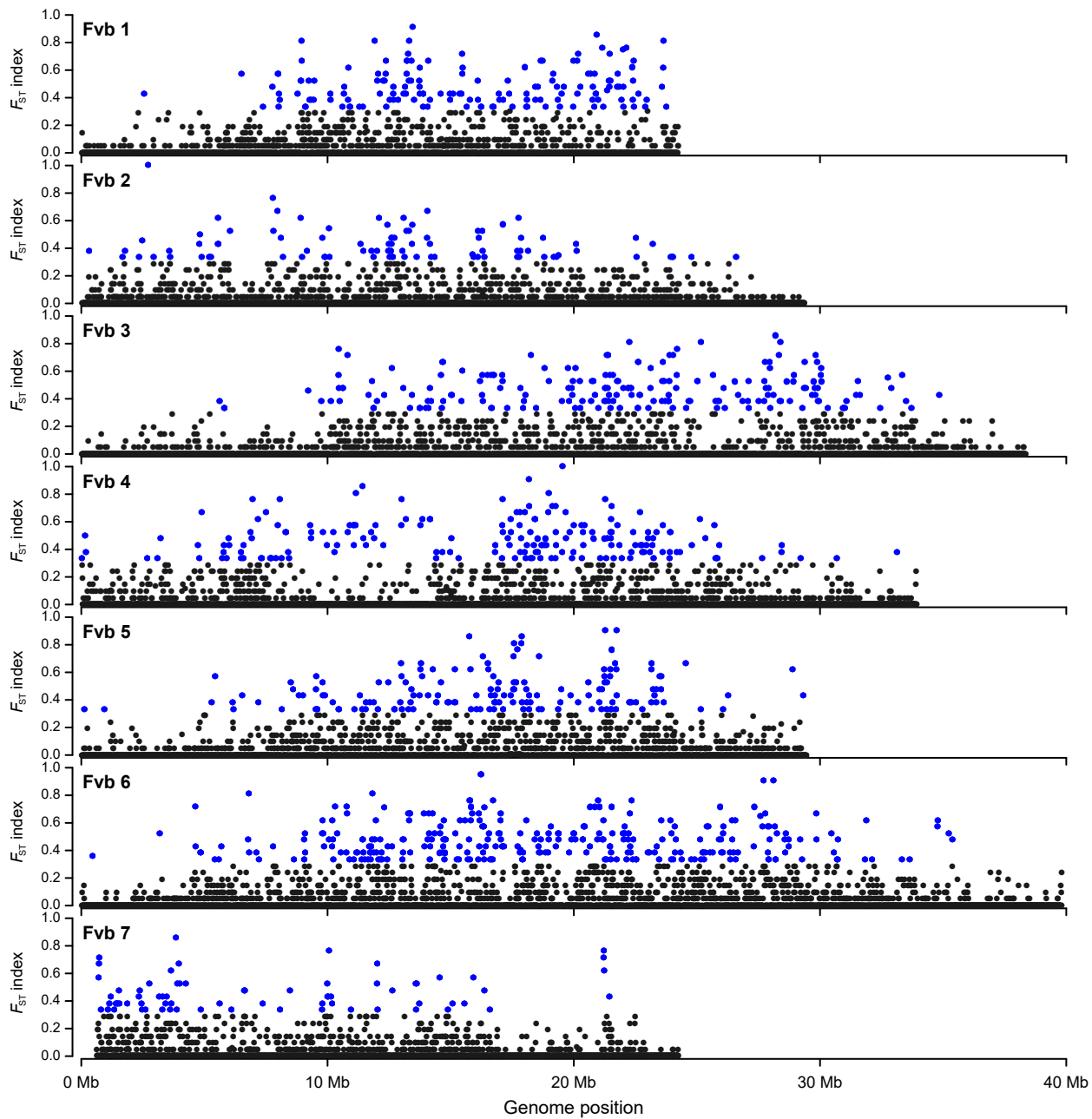

Supplement: msad049_Supplementary_Data [file msad049_supplementary_data.zip › Figure S8_Genome distribution of high Fst regions.pdf]

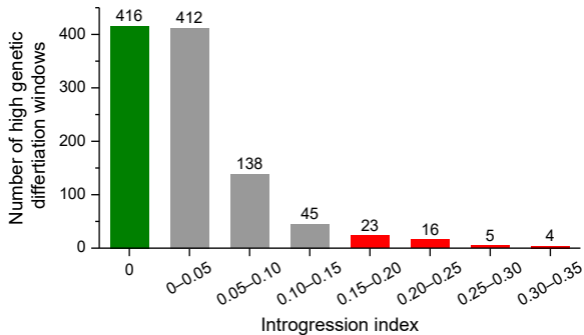

Supplement: msad049_Supplementary_Data [file msad049_supplementary_data.zip › Figure S9_The overlap between regions of high genetic differentiation and different introgression.pdf]

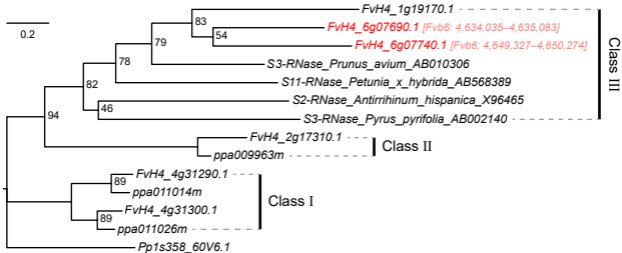

Supplement: msad049_Supplementary_Data [file msad049_supplementary_data.zip › Figure S10. ML tree of S-RNase-like genes.pdf]
